# Psychometric dataset of the Indonesian adaptation of the adolescent peer relations instrument (APRI) bullying perpetration subscale among Indonesian senior high school students

**DOI:** 10.1016/j.dib.2026.113065

**Published:** 2026-07-08

**Authors:** Lutfi Arya, Anwar Fadlil Abdullah, Budi Sulaeman, Rini Nurahaju, Wiwin Dwi Handayani, Bagiyo Suwasono

**Affiliations:** a Psychology Department, Faculty of Psychology, Universitas Hang Tuah, Surabaya, East Java, Indonesia; b Doctoral Programme, Faculty of Psychology, Universitas Padjadjaran, Sumedang, West Java, Indonesia; c Psychology Department, Faculty of Humanities, Bina Nusantara University, Jakarta, DKI Jakarta, Indonesia; d Postgraduate Program of Marine Coastal Engineering, Universitas Hang Tuah, Surabaya, East Java, Indonesia

**Keywords:** Bullying perpetration, Adolescent peer relations instrument (APRI), High school, Confirmatory factor analysis, Reliability analysis

## Abstract

Data were collected from 465 high school students in one city in Indonesia using the Adolescent Peer Relations Instrument (APRI) Bullying Perpetration Subscale, adapted into Indonesian. The instrument was translated and culturally adapted through international guidelines, including forward translation, backward translation, and expert review. The available dataset includes responses to 18 self-report items that measure bullying behavior in perpetrators related to verbal, social, and physical dimensions. Additionally, this data includes demographic data consisting of gender. The dataset includes statistical results from psychometric evaluation procedures. Confirmatory Factor Analysis (CFA) is used to check the factor structure. The model fit is assessed using Chi-square (χ²), Root Mean Square Error of Approximation (RMSEA), Comparative Fit Index (CFI), and Standardized Root Mean Square Residual (SRMR). Reliability estimates include Cronbach's alpha (α). This dataset can be reused for cross-cultural validation studies, psychometric comparison research, structural equation modeling applications, and secondary analyses that examine bullying behavior among adolescents. This data can also support methodological research on adaptation and validation of instruments within a non-Western context.

Specifications Table

**Table:** 

Subject	Social Science
Specific subject area	Quantitative Psychology
Type of data	Type of data: Table and Comma Separated Values (CSV) Data formats: Raw and Processed
Data collection	Data were collected via an online questionnaire administered to Indonesian senior high school students (Grades 10–12) actively enrolled at the time of data collection. The bullying perpetration subscale was adapted from the Adolescent Peer Relations Instrument (APRI) as documented in the CDC compendium [1], originally developed by Parada [2]., translated using forward–backward procedures and expert review. Inclusion required active enrollment and informed consent. Incomplete or invariant responses were excluded. CFA and reliability analyses were conducted using JASP.
Data source location	Institution: Universitas Hang Tuah City/Town/Region: Surabaya Country: Indonesia
Data accessibility	Repository name: Mendeley Data Data identification number: 10.17632/v9y9rtzsf9.2 Direct URL to data: https://data.mendeley.com/datasets/v9y9rtzsf9/2 Instructions for accessing these data: Publicly available; includes raw and processed .csv files, compiled .xlsx file, and .jasp file for reproducibility.
Related research article	None

## 1 Value of the Data

• These data provide raw responses to the Indonesian-adapted Bullying Perpetration Subscale from senior high school students, enabling secondary psychometric analyses such as reliability estimation and confirmatory factor analysis
• The dataset includes separate files for physical, verbal, and social bullying dimensions, facilitating dimension-specific analyses, especially reliability analysis
• The availability of raw data, processed datasets, and the original JASP analysis file [*3*] enables independent verification, replication, and re-analysis using alternative statistical software or modeling approaches.
• The documentation of the Indonesian adaptation process supports reuse for cross-cultural comparison, measurement invariance testing, and scale validation, while also serving as an evidence base for policymakers and schools in designing and evaluating anti-bullying interventions.
• The dataset contributes to addressing a methodological gap in Indonesia, where psychometrically validated, unidimensional measures of bullying perpetration appear to remain limited. Available national reports from the Indonesian Child Protection Commission have largely focused on complaints and victim-related cases, while systematic data on bullying perpetration remain comparatively limited.

## 2 Background

The dataset was generated within a preregistered project to document the Indonesian adaptation of the Bullying Perpetration Subscale for senior high school students. The APRI was originally developed to assess bullying behaviors across physical, verbal, and social dimensions among adolescents. Given the need for culturally appropriate measurement tools in non-Western educational contexts, the instrument was translated and adapted for use in Indonesia, following standardized cross-cultural adaptation procedures.

Data were collected to document item-level responses and to examine psychometric characteristics, including internal consistency coefficients (Cronbach’s alpha) and factorial structure evaluated through confirmatory factor analysis (CFA) using JASP software.

This data article provides access to the raw and processed datasets, as well as the associated analysis files, enabling independent verification and secondary methodological examination beyond any related research publication.

## 3 Data Description

This dataset contains responses from senior high school adolescents who completed the Indonesian adaptation of the perpetrator subscale of the APRI. Data were collected through an online survey platform and analyzed using sa for psychometric evaluation.

The repository includes:

• Raw Data (APRI_Raw Data): anonymized item-level responses and demographic information (gender), with each row representing one participant.
• Item Translation (APRI_ScaleItem): documentation of the forward–backward translation process, including original and Indonesian versions of the instructions and items.
• Confirmatory Factor Analysis Result (APRI_Confirmatory Factor Analysis.csv): CFA output including standardized factor loadings and model fit indices (χ², RMSEA, CFI, TLI, SRMR).
• Unidimensional Reliability (APRI_Unidimensional Reliability.csv): internal consistency analysis for the overall construct using Cronbach’s α.
• Verbal, Social, and Physical Aspect Reliability Files: reliability outputs for each APRI perpetrator dimension, including internal consistency coefficients and item-level statistics.
• Analysis File (APRI_JASP.jasp): complete statistical procedures and outputs to ensure transparency and reproducibility.

The APRI perpetrator subscale comprises three dimensions: Verbal Aggression, Social Aggression, and Physical Aggression. Each response record represents one participant. No personally identifiable information is included in the dataset.

To provide an overview of the psychometric properties of the adapted instrument, the main confirmatory factor analysis (CFA) fit indices are presented in Table 1. Reliability coefficients for each dimension and the total scale are summarized in Table 2. Full item-level statistics and detailed outputs are available in the repository files.

**Table 1:** Model fit indices (CFA).

χ²	df	CFI	TLI	RMSEA	SRMR
278.660	132	.972	.967	.049	.07

**Table 2:** Reliability coefficients.

Dimension	Cronbach’s α
Verbal Aggression	.606
Social Aggression	.638
Physical Aggression	.804
Total Scale	.822

All raw data, reliability coefficients, and model fit indices are preserved within the JASP file (APRI_JASP.jasp) to ensure full transparency and reproducibility of the analyses.

## 4 Experimental Design, Materials and Methods

Data were collected via an online survey to evaluate the psychometric characteristics of the Indonesian version of the perpetrator subscale. The questionnaire was administered using Google Forms and distributed through social media platforms using a convenience sampling approach. Participants first reviewed an electronic informed consent form stating that participation was voluntary, responses were anonymous, no personally identifiable information was collected, and withdrawal was possible at any time. Only participants who provided consent could access the questionnaire.

Eligible participants were adolescents actively enrolled in senior high school (or equivalent) within the target age range of the original APRI development. Based on the recommended ratio of 5 to 10 respondents per indicator [4], the minimum required sample size for the 18-item instrument ranged from 90 to 180 respondents. A total of 476 responses were collected, of which responses were excluded due to missing responses (n = 2), invariant or inattentive response patterns (straight-lining responses; n = 4), or unverifiable school enrollment status (n = 5), resulting in a final analytic sample of 465.

The adapted instrument consisted of three aspects: verbal (e.g., "Teased them by saying things to them"), physical (e.g., "Pushed or shoved a student"), and social bullying (e.g., "Got other students to start a rumor about a student"), with each aspect represented by six items, resulting in a total of 18 self-report items rated on a seven-point frequency scale (0 = never to 6 = every day). The adaptation process involved forward and backward translation, as well as expert review. Gender was collected as demographic information.

Data were exported in .csv format. Analyses were conducted using JASP (version 0.16.2). Confirmatory factor analysis (CFA) was performed to estimate model fit indices (χ², df, RMSEA, CFI, TLI, SRMR). Internal consistency was calculated using Cronbach’s α. The complete analytical procedures are preserved in the accompanying .jasp file.

## Limitations

Data collection for this dataset was conducted in a single city in Indonesia, thus limiting its geographic coverage. Furthermore, this data was collected online, requiring internet access and potentially limiting the reach of students without it. This dataset is self-reported; no external behavioral validation was conducted. The only demographic data available is gender; no other information was added for additional demographic analysis. Furthermore, the data collection is cross-sectional rather than longitudinal, which would allow for more concrete behavioral consistency.

## Ethics Statement

The ethical measures implemented in this study adhered to the principles of the Declaration of Helsinki [*5*]. Prior to data collection, a formal cooperation agreement was established between the Bullying Crisis Center (BCC), Faculty of Psychology, Universitas Hang Tuah, and the participating school, which served as institutional authorization. Written informed consent was obtained from all participants before participation. Participation was entirely voluntary and without financial compensation. All participant data were immediately anonymized using sequential codes (S-001 to S-N), with no personally identifiable information retained in the dataset.

## CRediT Author Statement

**Lutfi Arya:** Conceptualization, Investigation, Resources, Funding acquisition, Writing - Review & Editing. **Anwar Fadlil Abdullah:** Methodology, Formal analysis, Data Curation, Writing - Original Draft. **Budi Sulaeman:** Project administration, Validation, Visualization, Writing - Review & Editing. **Rini Nurahaju**: Literature Review, Visualization, Writing – Review & Editing. **Wiwin Dwi Handayani**: Data Collection, Investigation, Resources. **Bagiyo Suwasono**: Supervision, Funding Acquisition, Project Administration.

## Declaration of Generative AI and AI-Assisted Technologies in the Manuscript Preparation Process

During the preparation of this work the author(s) used ChatGPT by OpenAI in order to improve sentence structure, enhance academic writing clarity, and refine the language of the manuscript. After using this tool/service, the author(s) reviewed and edited the content as needed and take(s) full responsibility for the content of the published article.

## Declaration of Competing Interest

The authors declare that they have no known competing financial interests or personal relationships that could have appeared to influence the work reported in this paper.

## Data Availability

Mendeley DataDataset of the Indonesian Adaptation of the Adolescent Peer Relations Instrument (APRI) Bullying Perpetration Subscale among Indonesian Senior High School Students (Original data)

## Appendix Supplementary materials

Image, application 1

Image, application 2

Image, application 3
