# Supplementary material for: Psychometric dataset of the Indonesian adaptation of the adolescent peer relations instrument (APRI) bullying perpetration subscale among Indonesian senior high school students
Source: Data Brief. 2026 Jul 8;67:113065. doi: 10.1016/j.dib.2026.113065 (PMC13382648; doi:10.1016/j.dib.2026.113065)
Supplement: Supplementary file 1 [file mmc1.pdf]

ETHICS STATEMENT

Associated to the Article:

Psychometric Dataset of the Indonesian Adaptation of the Adolescent Peer Relations Instrument (APRI) Bullying Perpetration Subscale among Indonesian Senior High School Students

1. Overview

This study was conducted by the Bullying Crisis Center (BCC), Faculty of Psychology, Universitas Hang Tuah Surabaya, as part of an ongoing research initiative on the prevalence of bullying among Indonesian senior high school students. Although formal review by an Institutional Review Board (IRB) was not required under the institutional framework applicable at the time of data collection, the research team adhered to internationally recognized ethical principles for research involving human participants, including the Declaration of Helsinki. The following sections describe the specific ethical measures implemented.

2. Institutional Authorization

Prior to data collection, a formal cooperation agreement was established between the Bullying Crisis Center (BCC), Faculty of Psychology, Universitas Hang Tuah Surabaya, and the participating school in Surabaya, Indonesia. This agreement was documented in a written research proposal submitted to and accepted by the school administration. The proposal outlined the purpose of the study, the data collection procedure, data handling practices, and the benefits to be returned to the institution.

Under the terms of this agreement, the school administration granted permission for the research team to administer the questionnaire to its students. The school retained the right to receive a full summary report of the findings and to remain anonymous in any subsequent publication.

No ethics committee approval number was issued in this context; institutional authorization was granted directly by the school, as the responsible authority for its student population.

3. Informed Consent

Written informed consent was obtained from all participants prior to data collection. The consent form was developed in accordance with the principles of the Declaration of Helsinki and included the following elements:

- A clear description of the purpose and nature of the study.
- A statement that participation was entirely voluntary and that participants could withdraw at any time without penalty.
- An explanation of how data would be collected, stored, and used.
- Assurance that all responses would be kept confidential and that no personally identifiable information would be disclosed.
- Contact information of the research team for any questions or concerns.

Because participants were minors (senior high school students aged 15–18 years), school-level institutional authorization served as the overarching consent mechanism, consistent with practices for school-based research in Indonesia.

4. Confidentiality and Anonymity

All participant data were anonymized immediately upon collection. Each participant was assigned a sequential alphanumeric code (S-001, S-002, ..., S-N), and no names or other personally identifiable information were retained in the dataset. The school's identity was similarly protected in this publication in accordance with the cooperation agreement.

5. Risk Assessment and Benefit to Participants

The study used only a self-report questionnaire to measure bullying perpetration. No experimental manipulation, deception, physiological measurement, or invasive procedure was employed. The risk of harm to participants was therefore assessed as minimal.

As a direct benefit to the participating institution, the research team provided the school administration with a full written report of the findings upon completion of the study. This report included prevalence estimates, breakdowns by bullying type and gender, and evidence-based recommendations for prevention and intervention programs. This reciprocal arrangement is consistent with the ethical principle of beneficence toward the communities involved in research.

6. Ethical Compliance Summary

The table below summarizes the ethical safeguards implemented in this study.

Ethical Compliance Checklist		
**Status**	**Ethical Requirement**	**How This Study Complies**
✓	**Institutional Authorization**	Formal cooperation agreement obtained from the school institution in Surabaya prior to data collection, facilitated through Bullying Crisis Center (BCC), Faculty of Psychology, Universitas Hang Tuah Surabaya.
✓	**Informed Consent**	Written informed consent was obtained from all participants before participation, in accordance with the principles of the Declaration of Helsinki.
✓	**Voluntary Participation**	Participation was entirely voluntary. Students were explicitly informed of their right to withdraw at any time without consequence.
✓	**Confidentiality & Anonymity**	Participant identities were anonymized using sequential codes (S-001 to S-N). No personally identifiable information was retained in the dataset.
✓	**Risk Minimization**	The study involved only a self-report questionnaire. No experimental manipulation, deception, or invasive procedure was conducted.
✓	**Benefit to Institution**	A full research report summarizing bullying prevalence findings was provided to the school as agreed. The school retains the right to keep its identity confidential in publications.
✓	**Minor Participants**	All participants were senior high school students (aged 15–18). Consent procedures adhered to guidelines for research involving minors, and school authorization served as the institutional safeguard.

7. Author Declaration

The authors declare that all research procedures were conducted in compliance with the ethical standards described above, consistent with the principles of the Declaration of Helsinki (World Medical Association, 2013). The study did not require formal IRB approval under the applicable institutional guidelines. All participants provided written informed consent. Data were collected, stored, and reported in a manner that protects participant confidentiality.

Author:
Lutfi Arya, M.Psi, Psikolog

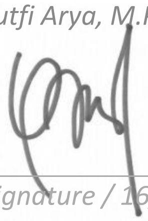

Signature / 16/05.2026

Institution:
Bullying Crisis Center (BCC)
Faculty of Psychology
Universitas Hang Tuah Surabaya

Reference

World Medical Association. (2013). World Medical Association Declaration of Helsinki: Ethical principles for medical research involving human subjects. *JAMA*, 310(20), 2191–2194. https://doi.org/10.1001/jama.2013.281053
