# Supplementary material for: Psychometric dataset of the Indonesian adaptation of the adolescent peer relations instrument (APRI) bullying perpetration subscale among Indonesian senior high school students
Source: Data Brief. 2026 Jul 8;67:113065. doi: 10.1016/j.dib.2026.113065 (PMC13382648; doi:10.1016/j.dib.2026.113065)
Supplement: Supplementary file 2 [file mmc2.pdf]

Complete Questionnaire (English Version)

Adolescent Peer Relations Instrument (APRI) — Bullying Perpetration Subscale

Indonesian Adaptation for Senior High School Students

Instructions

Since you have been at this school THIS YEAR how often HAVE YOU done any of the following things to a STUDENT (or students) at this school. CIRCLE THE NUMBER THAT IS CLOSEST TO YOUR ANSWER.

Response Scale

Please rate each item using the following scale:

Value	Description
0	Never
1	Sometimes
2	Once or twice a month
3	Once a week
4	Several times a week
5	Everyday

Questionnaire Items

APRI Bullying Perpetration Subscale — Items				
No.	Dimension	Item	Reverse-Scored	Score
1	Verbal bullying	Teased them by saying things to them	No	0–5
2	Physical bullying	Pushed or shoved a student	No	0–5
3	Verbal bullying	Made rude remarks at a student	No	0–5
4	Social bullying	Got my friends to turn against a student	No	0–5
5	Verbal bullying	Made jokes about a student	No	0–5

6	Physical bullying	Crashed into a student on purpose as they walked by	No	0–5
7	Verbal bullying	Picked on a student by swearing at them	No	0–5
8	Social bullying	Told my friends things about a student to get them into trouble	No	0–5
9	Physical bullying	Got into a physical fight with a student because I didn't like them	No	0–5
10	Verbal bullying	Said things about their looks they didn't like	No	0–5
11	Social bullying	Got other students to start a rumor about a student	No	0–5
12	Physical bullying	I slapped or punched a student	No	0–5
13	Social bullying	Got other students to ignore a student	No	0–5
14	Verbal bullying	Made fun of a student by calling them names	No	0–5
15	Physical bullying	Threw something at a student to hit them	No	0–5
16	Physical bullying	Threatened to physically hurt or harm a student	No	0–5
17	Social bullying	Left them out of activities or games on purpose	No	0–5
18	Social bullying	Kept a student away from me by giving them mean looks	No	0–5

Scoring Note

All 18 items are scored in the same direction (0–5). No items are reverse-scored. Higher total scores indicate greater frequency of bullying perpetration behavior.

Total score range: 0–90

Reference: R.H. Parada, Adolescent Peer Relations Instrument: *A Theoretical and Empirical Basis for the Measurement of Participant Roles in Bullying and Victimization of Adolescence: An Interim Test Manual and a Research Monograph: A Test Manual*, Publication Unit, Self-concept Enhancement and Learning Facilitation (SELF) Research Centre, University of Western Sydney, Penrith South, DC, Australia, 2000.
