# Supplementary material for: Psychometric dataset of the Indonesian adaptation of the adolescent peer relations instrument (APRI) bullying perpetration subscale among Indonesian senior high school students
Source: Data Brief. 2026 Jul 8;67:113065. doi: 10.1016/j.dib.2026.113065 (PMC13382648; doi:10.1016/j.dib.2026.113065)
Supplement: Supplementary file 3 [file mmc3.pdf]

Data in Brief
Open access

CODEBOOK

ASSOCIATED TO THE ARTICLE:

Psychometric Dataset of the Indonesian Adaptation of the Adolescent Peer Relations Instrument (APRI) Bullying Perpetration Subscale among Indonesian Senior High School Students

Author:

Lutfi Arya*[1,2], Anwar Fadlil Abdullah[2], Budi Sulaeman[2,3], Rini Nurahaju[1], Wiwin Dwi Handayani[1], Bagiyo Suwasono[4]

[1]*Psychology Department, Faculty of Psychology, Universitas Hang Tuah, Surabaya, East Java, Indonesia*

[2]*Doctoral Programme, Faculty of Psychology, Universitas Padjadjaran, Sumedang, West Java, Indonesia*

[3]*Psychology Department, Faculty of Humanities, Bina Nusantara University, Jakarta, DKI Jakarta, Indonesia*

[4]*Postgraduate Program of Marine Coastal Engineering, Hang Tuah University, Surabaya, Indonesia*

Table 1 presents a Codebook of the variables included in the data file: the demographic variables, the APRI Bullying Perpetration Subscale items, and computed scores. The variable names in the data file are provided in the 'Variable Name in Data File' column. The response format is included in the 'Item' column. The complete reference for the source scale can be found at the end of this document.

Table 1.

Measured Variables in the Data File: Items, Variable Names, and Sources.

Variable	Item	Variable Name in Data File	Source
**Section 1: Demographic Variables**			
**Participant ID**	Unique participant identifier assigned sequentially.<br>Format: S-001 to S-N	ID Subject	—
**Gender**	Statement: What is your gender?<br>(1) Laki-laki [Male]<br>(2) Perempuan [Female]	Sex	—
**Section 2: APRI Bullying Perpetration Subscale Items (Response scale: 0 = Never; 1 = Sometimes; 2 = Once or twice a month; 3 = Once a week; 4 = Several times a week; 5 = Everyday)**			
Item 1 — Verbal bullying	1. Teased them by saying things to them	V1	[1]

Data in Brief
Open access

Variable	Item	Variable Name in Data File	Source
	(0) Never / (1) Sometimes / (2) Once or twice a month / (3) Once a week / (4) Several times a week / (5) Everyday		
Item 2 — Physical bullying	2. Pushed or shoved a student (0) Never / (1) Sometimes / (2) Once or twice a month / (3) Once a week / (4) Several times a week / (5) Everyday	V2	[1]
Item 3 — Verbal bullying	3. Made rude remarks at a student (0) Never / (1) Sometimes / (2) Once or twice a month / (3) Once a week / (4) Several times a week / (5) Everyday	V3	[1]
Item 4 — Social bullying	4. Got my friends to turn against a student (0) Never / (1) Sometimes / (2) Once or twice a month / (3) Once a week / (4) Several times a week / (5) Everyday	V4	[1]
Item 5 — Verbal bullying	5. Made jokes about a student (0) Never / (1) Sometimes / (2) Once or twice a month / (3) Once a week / (4) Several times a week / (5) Everyday	V5	[1]
Item 6 — Physical bullying	6. Crashed into a student on purpose as they walked by (0) Never / (1) Sometimes / (2) Once or twice a month / (3) Once a week / (4) Several times a week / (5) Everyday	V6	[1]
Item 7 — Verbal bullying	7. Picked on a student by swearing at them (0) Never / (1) Sometimes / (2) Once or twice a month / (3) Once a week / (4) Several times a week / (5) Everyday	V7	[1]
Item 8 — Social bullying	8. Told my friends things about a student to get them into trouble (0) Never / (1) Sometimes / (2) Once or twice a month / (3) Once a week / (4) Several times a week / (5) Everyday	V8	[1]
Item 9 — Physical bullying	9. Got into a physical fight with a student because I didn't like them (0) Never / (1) Sometimes / (2) Once or twice a month / (3) Once a week / (4) Several times a week / (5) Everyday	V9	[1]
Item 10 — Verbal bullying	10. Said things about their looks they didn't like (0) Never / (1) Sometimes / (2) Once or twice a month / (3) Once a week / (4) Several times a week / (5) Everyday	V10	[1]

Variable	Item	Variable Name in Data File	Source
Item 11 — Social bullying	11. Got other students to start a rumor about a student (0) Never / (1) Sometimes / (2) Once or twice a month / (3) Once a week / (4) Several times a week / (5) Everyday	V11	[1]
Item 12 — Physical bullying	12. I slapped or punched a student (0) Never / (1) Sometimes / (2) Once or twice a month / (3) Once a week / (4) Several times a week / (5) Everyday	V12	[1]
Item 13 — Social bullying	13. Got other students to ignore a student (0) Never / (1) Sometimes / (2) Once or twice a month / (3) Once a week / (4) Several times a week / (5) Everyday	V13	[1]
Item 14 — Verbal bullying	14. Made fun of a student by calling them names (0) Never / (1) Sometimes / (2) Once or twice a month / (3) Once a week / (4) Several times a week / (5) Everyday	V14	[1]
Item 15 — Physical bullying	15. Threw something at a student to hit them (0) Never / (1) Sometimes / (2) Once or twice a month / (3) Once a week / (4) Several times a week / (5) Everyday	V15	[1]
Item 16 — Physical bullying	16. Threatened to physically hurt or harm a student (0) Never / (1) Sometimes / (2) Once or twice a month / (3) Once a week / (4) Several times a week / (5) Everyday	V16	[1]
Item 17 — Social bullying	17. Left them out of activities or games on purpose (0) Never / (1) Sometimes / (2) Once or twice a month / (3) Once a week / (4) Several times a week / (5) Everyday	V17	[1]
Item 18 — Social bullying	18. Kept a student away from me by giving them mean looks (0) Never / (1) Sometimes / (2) Once or twice a month / (3) Once a week / (4) Several times a week / (5) Everyday	V18	[1]
**Section 3: Computed Scores**			
**Total Perpetration Score**	Sum of all 18 APRI Bullying Perpetration items. Range: 0–90. No items are reverse-scored.	Total Pelaku	[1]

Variable	Item	Variable Name in Data File	Source
	Higher scores indicate greater bullying perpetration frequency.		
**Verbal Bullying Subscale Score**	Sum of verbal bullying items (Items 1, 3, 5, 7, 10, 14).<br>Range: 0–30.	Verbal	[1]
**Social Bullying Subscale Score**	Sum of social bullying items (Items 4, 8, 11, 13, 17, 18).<br>Range: 0–30.	Sosial	[1]
**Physical Bullying Subscale Score**	Sum of physical bullying items (Items 2, 6, 9, 12, 15, 16).<br>Range: 0–30.	Fisik	[1]
**Perpetration Category**	Categorical classification of perpetration frequency based on the mean item score. Derived from the response scale labels:<br>(0) Tidak Pernah [Never]<br>(1) Terkadang [Sometimes]<br>(2) Sekali dalam sebulan [Once or twice a month]<br>(3) Sekali dalam seminggu [Once a week]<br>(4) Beberapa kali dalam seminggu [Several times a week]<br>(5) Setiap hari [Everyday]	Kategori Pelaku	[1]
**Mean Score**	Mean of all 18 APRI Bullying Perpetration items.<br>Range: 0–5. Computed as Total Pelaku / 18.	Rata2	[1]

Note. Items 1, 3, 5, 7, 10, 14 = Verbal bullying dimension; Items 4, 8, 11, 13, 17, 18 = Social bullying dimension; Items 2, 6, 9, 12, 15, 16 = Physical bullying dimension. No items are reverse-scored.

References

[1] } R.H. Parada, *Adolescent Peer Relations Instrument: A Theoretical and Empirical Basis for the Measurement of Participant Roles in Bullying and Victimization of Adolescence: An Interim Test Manual and a Research Monograph: A Test Manual*, Publication Unit, Self-concept Enhancement and Learning Facilitation (SELF) Research Centre, University of Western Sydney, Penrith South, DC, Australia, 2000.
